# Delusions

**Published:** 1908-08-08

**Authors:** 


### Mental Diseases.

### DELUSIONS.

A delusion, as was remarked in a former article, is a false belief, and is a purely mental process. Whether, however, a false belief is an *insane* delusion is at times a difficult matter to decide. The view generally held as to what constitutes an insane delusion is that it must be, (1) incompatible with the education and experience of the individual, and (2) unassailable by logical argument. A point is always reached in arguing with a deluded insane patient where he leaves the subject altogether or goes off at a tangent. The patient who believes he is the Almighty, and says he can call down a legion of angels to do his bidding, will ask one to open the door for him; and when it is pointed out to him that this is needless since he can command the angels to do it, will not only refuse to acknowledge the justice of the remark, but will say that that is not the point.

The cause of delusions is often obscure, and at times cannot be ascertained at all. They may be due to hallucinations, or morbid alteration in subjective sensations. Delusions of this kind are seen in ideas of persecution due to hallucinations of some special sense; and the large delusions of certain general paralytics as to their great personal achievements, powers, wealth, etc., are due, probably, to exaggerated sense of well being. They may, again, be due to imagination or suggestion, whilst reason and judgment are temporarily in abeyance, as in the rapidly changing delusions of acute mania; or to strong emotional states, as seen in some delusions of a religious character. Defect of memory also, sometimes plays a part in causation, as may be noticed in some delusions of identity, and also in those delusions of the epileptic who, having no recollection of the fit, attributes his bodily soreness to brutal treatment by his attendants. New delusions are very rare, either inside or outside asylums, unless some new scientific discovery, such as *x*-rays, wireless telegraphy, or radium gives rise to a fresh crop of them.

The main divisions of delusions are (1) temporary or changing, and (2) fixed. Temporary delusions are well seen in acute mania, in which they often change with every passing fancy or sensation of the patient. They are of less importance the more quickly they change, and will probably totally disappear as the acute symptoms subside. Fixed delusions, which persist unaltered for long periods, are of grave import and seriously affect the prognosis. The longer a delusion persists, the less likelihood is there of it ever being eradicated, and the more danger of the case passing into a chronic insanity.

Delusions, besides occurring as one of the symptoms of mental disease, may be the only cause that can be found for the insane conduct and conversation of the individual. Certain cases of pure delusional insanity are met with which are of gradual onset and which at no period display acute symptoms. The patient at no point in the development of the insanity displays morbid exaltation or depression. Hallucinations may, and probably do, occur in most of these cases, though they are sometimes impossible to detect. All that is noticed is that delusions gradually appear, persist, and become fixed. In the early stages of this form the disease is said to be unsystematised, but later, when the delusions have been, so to speak, worked up and made less crude, have had details filled in, and have had essentials, overlooked at first, added to them, then it is said to be systematised. Delusions of persecution are very common in this form of insanity. In one case known to the writer the patient, an educated lady, believed herself to be descended from King Charles II. and to be entitled to property from that monarch and from Catherine of Braganza. She had never had any sign of depression, nor of excitement beyond ordinary quick temper, nor any discoverable hallucinations. Her delusions, when she first came under care, were systematised in a high degree. She had carefully studied the history and genealogy of the person she imagined herself to be, and had even been to Portugal for details. She could not be tripped in historical accuracy, and was quite beyond logical argument as to the obvious fallacy of her belief. Owing to her delusions her conduct finally brought her into conflict with the police. Cases such as this, displaying neither acute symptoms nor hallucinations, are rare, and when they occur are generally troublesome and should be certified.

The most important point that occurs in dealing with a deluded patient is the question of certification. The danger of deluded states arises from the fact that they may lead the patient to suicide, homicide, crime, etc., and if the delusions are of such a nature as to influence the patient's conduct so that he may become dangerous, morally or physically, to himself or others, certification should be advised at once. The fact that the belief, though obviously false to everyone else, is absolutely true to the patient, makes logical argument of no avail in removing it; indeed, it is one of the first principles in dealing with these cases never to argue with them. The only method of treatment that can be adopted is to endeavour to correct the cause of the delusions, if such cause can be discovered.

